# IGFBP2 is a biomarker for predicting longitudinal deterioration in renal function in type 2 diabetes

**DOI:** 10.1530/EC-12-0053

**Published:** 2012-10-24

**Authors:** Ram P Narayanan, Bo Fu, Adrian H Heald, Kirk W Siddals, Robert L Oliver, Julie E Hudson, Antony Payton, Simon G Anderson, Anne White, William E R Ollier, J Martin Gibson

**Affiliations:** 1 Vascular Research Group The University of Manchester Manchester, M13 9PT UK; 2 School of Community Based Medicine, The University of Manchester Manchester, M13 9PT UK; 3 Centre for Integrated Genomic Medical Research, The University of Manchester Manchester, M13 9PT UK; 4 Cardiovascular Research Group The University of Manchester Manchester, M13 9PT UK; 5 Endocrinology and Diabetes, Faculty of Medical, Human and Life Sciences The University of Manchester Manchester, M13 9PT UK; 6 Salford R&D, Salford Royal Hospital NHS Foundation Trust Salford, M6 8HD UK; 7 Department of Endocrinology and Diabetes Salford Royal Hospital NHS Foundation Trust Salford, M6 8HDUK

**Keywords:** IGFBP2, longitudinal trends, renal function, real-world data

## Abstract

**Objective:**

Insulin-like growth factors are implicated in the development of diabetic nephropathy. IGF-binding protein 2 (IGFBP2) and IGF2 are expressed in the kidney, but their associations with diabetic nephropathy are unclear. We therefore tested the hypothesis that circulating levels of IGF2 and IGFBP2 predict longitudinal renal function in individuals with type 2 diabetes.

**Design and methods:**

IGFBP2 and IGF2 measurements were performed in 436 individuals (263 males) with type 2 diabetes. Linear mixed-effect regression analysis was used to model the relationship between plasma IGFBP2 concentration and longitudinal changes in estimated glomerular filtration rate (eGFR) over an 8-year period. Analyses were also performed for IGF1, IGF2, IGFBP1 and IGFBP3 concentrations as predictors of longitudinal renal outcomes.

**Results:**

High IGFBP2 concentration at baseline was associated with a decreased eGFR over an 8-year period (*β*=−0.02, (95% confidence interval −0.03 to −0.01), *P*<0.001). High IGFBP1, IGFBP2 and IGFBP3 were also associated with low baseline eGFR concentration.

**Conclusion:**

This study demonstrates that IGFBP2 is a predictor of longitudinal deterioration of renal function in type 2 diabetes.

## Introduction

The rising prevalence of diabetes worldwide is associated with increased morbidity and mortality (1). Diabetic nephropathy is a major complication of diabetes and is the commonest cause of end-stage renal disease (2). The development and progression of diabetic kidney disease is influenced by a number of biological factors. Among these, the insulin-like growth factor (IGF) family of proteins have been repeatedly implicated in the development of nephropathy and other complications of diabetes.

The IGF system comprises the two primary ligands, namely IGF1 and IGF2, six high-affinity fully characterised IGF-binding proteins (IGFBP1–IGFBP6) and the IGF receptors.

IGF1 and IGF2 are anabolic peptides with extensive structural and functional homology with insulin. They are ubiquitously expressed and play an indispensible role in the control of growth, proliferation and metabolism. IGF1 and IGF2 effect local and systemic responses through autocrine, paracrine and endocrine mechanisms. Both IGFs are expressed in the kidney, and rodent models have demonstrated renal expression of all six IGFBPs (3, 4).

A number of renal and systemic perturbations of the IGF system are known to occur in diabetic nephropathy (5). It has been speculated that IGF1-mediated increases of nitric oxide synthesis contribute to glomerular hyperfiltration in the early stages of diabetic kidney disease (6). IGF1 accumulates in the kidney soon after streptozotocin (STZ)-induced diabetes in rats and mice, but renal IGF1 expression is not usually increased, suggesting that the increased IGF1 is derived from the systemic circulation (7, 8). In contrast, both IGFBP1 renal expression and protein levels are elevated in mouse models of type 1 diabetes (8). One hypothesis is that in diabetic kidney disease there is an IGF-independent primary increase in IGFBP1 concentration in the kidney that subsequently traps IGF1 within the renal glomerulus. The sequestered IGF1 induces the glomerular hypertrophy and renal hyperfiltration that is characteristic of early diabetic nephropathy (9, 10).

Research with respect to the interactions of IGF2 in diabetic kidney disease has been limited. IGFBP2 has preferential affinity for IGF2 and is also widely expressed in the renal glomerulus (3). In this study, we used ‘real-world’ data to examine the hypothesis that concentrations of IGF2 and IGFBP2 at baseline were associated with longitudinal trends in glomerular filtration rate (GFR) in type 2 diabetes.

## Subjects and methods

### Study population

Four hundred forty-five individuals with type 2 diabetes were recruited from the city of Salford in north-west England. The city has ∼9100 individuals with type 2 diabetes, and a representative sample was recruited from this background population for the Salford Longitudinal Diabetes Cohort. Approval was granted for this study by the Local Research Ethics Committee. Recruitment was undertaken in 2002–2003 from patients attending the diabetes outpatient clinics at the Salford Royal hospital for routine follow-up. Subjects who consented to participation donated a blood sample for circulating IGF protein measurements. Non-fasting blood samples were collected only at baseline. Access to longitudinal clinical measurements and drug prescription data was available from the Salford Integrated Records system which is a comprehensive linked electronic dataset of primary care and hospital data.

### Laboratory methods

Measurements of plasma IGF1, serum IGF2, plasma IGFBP1 (non-fasting), plasma IGFBP2 and plasma IGFBP3 were taken in the blood sample collected at study commencement.

IGF1 was measured using an Immulite 1000 immunoassay (Siemens, Camberley, UK) with an analytical sensitivity of 20 ng/ml and within and inter-assay coefficients of variation (CV) of <4.5 and <8.4% respectively. IGF2 was measured using an ELISA developed using antibodies that have been previously reported (11). The analytical sensitivity of the assay was <10 ng/ml. The intra-assay and inter-assay sensitivities were <6 and <10% respectively. IGFBP1 was measured using a locally developed assay that recognises all phosphoforms of IGFBP1 and has been reported in a previous study (12). This assay had inter-assay and intra-assay sensitivities of <15 and <9%, respectively, and the assay has an analytical sensitivity of 0.1 ng/ml. IGFBP2 measurements were performed using the commercial RayBio Human IGFBP2 ELISA kit (RayBiotech, Inc., Norcross, GA, USA) with a manufacturer reported sensitivity of <20 pg/ml. The intra-assay CV for this assay was <10% and the inter-assay CV was <12%. Plasma IGFBP3 was measured using the Immulite 1000 immunoassay that has an analytical sensitivity of 0.1 μg/ml. The intra-assay CV for this assay was <6% and the inter-assay CV was <10%.

Clinical and pharmacological data were obtained from a comprehensive linked database of primary and secondary care information related to the study patients. Measurements of serum creatinine, HbA1c, blood pressure, lipid profiles and all medication prescriptions for the years 2002–2009 were requested as a part of routine medical care by the subjects' health care providers. As all the study patients were recruited from the Salford area, the majority of blood tests was performed at the clinical laboratories of the local tertiary care hospital (Salford Royal NHS Foundation Trust). The last measurements of creatinine for each year between 2002 and 2009 in the health records were selected as representative for that year. Clinical and biochemical measurements for the year 2002 were used as baseline data.

Estimated GFR (eGFR) were calculated from serum creatinine measurements according to gender-specific modification of diet in renal disease (MDRD) equations (13). Serum creatinine measurements were performed using the Jaffe's method from 2002 to 2004 using the Roche Integra 700 Chemistry analyser, and from 2004 onwards using the Roche modular analyser. Changes in the diagnostic platforms for measurement of serum creatinine did not lead to changes in the normal reference ranges for these tests.

### Statistical analysis

Statistical analysis was conducted using the software package Stata 10SE (College Station, TX, USA). Comparison of IGF proteins as well as baseline HbA1c, blood pressure, total cholesterol, LDL cholesterol, high-density lipoprotein (HDL) cholesterol and body mass index (BMI) in individuals with or without baseline eGFR <60 ml/min per 1.73 m^2^ was made using independent samples *t*-tests. As measurements of IGF1, IGF2, IGFBP1, IGFBP2 and IGFBP3 were not normally distributed, their values were log-transformed for *t*-test purposes. All other variables studied followed a normal distribution.

Linear mixed-effect regression analysis was used to study the longitudinal trends in eGFR as outcomes of baseline serum IGF2 and plasma IGFBP2 concentrations, as well as plasma concentrations of other measured IGF proteins (IGF1, IGFBP1 and IGFBP3). Measurements of eGFR followed a normal distribution. The IGF proteins were studied as predictors of eGFR at baseline and longitudinally. In each analysis, these levels were adjusted for the following covariates defined *a priori*: age, gender, baseline measurements of all IGF proteins, baseline measurements of BMI, systolic blood pressure and HbA1c, duration of diabetes at study commencement and the use of angiotensin-converting enzyme (ACE) inhibitors and angiotensin receptor blockers during the study period. Longitudinal analyses were additionally adjusted for time interaction. Measurements of creatinine, HbA1c, body weight and blood pressure from the year 2002 were considered baseline measurements. Baseline eGFR measurements were available for all patients.

## Results

### The study population

Of the total 445 study subjects in the Salford Longitudinal Diabetes Cohort, 267 were of male gender (60%), and the mean age of the cohort was 62.8 years (95% confidence interval (95% CI) 61.8–63.8), 120 individuals had an eGFR <60 ml/min per 1.73 m^2^ at study commencement; including nine individuals with an eGFR <30 ml/min per 1.73 m^2^. These nine individuals with eGFR <30 ml/min per 1.73 m^2^ were excluded from further analyses, and the remaining 436 individuals were analysed in this study.

The background characteristics of the 436 individuals remaining in the study at study commencement (2002) and at the end of the study (2009) are described in Table 1. Two hundred ninety-six subjects were identified to have been prescribed ACE inhibitors or angiotensin receptor blockers during the study period (2002–2009). Mean circulating concentrations of the measured IGF proteins expressed as geometric mean (95% CIs) were as follows: IGF1, 125 ng/ml (120–130); IGF2, 569 ng/l (550–288); IGFBP1, 15 ng/ml (14–16); IGFBP2, 277 ng/ml (262–292) and IGFBP3, 4.2 mg/l (4.1–4.3).

Table 2 describes the baseline characteristics of subjects with eGFR above or below 60 ml/min per 1.73 m^2^ at study commencement. Three hundred twenty-five individuals had an eGFR >60 ml/min per 1.73 m^2^ (‘high eGFR’) while the remaining 111 individuals had an eGFR below 60 ml/min per 1.73 m^2^ (‘low eGFR’). Fifty-four (48.6%) of the ‘low eGFR’ individuals were male. Mean age of the ‘low eGFR’ group was higher than the ‘high eGFR’ group (*P*<0.0001), but interestingly the ‘low eGFR group’ demonstrated a lower HbA1c level (*P*=0.019) and a lower diastolic blood pressure (*P*=0.018). Mean measurements of BMI, systolic blood pressure, total cholesterol and HDL cholesterol were similar in the two groups.

**Figure 1:**
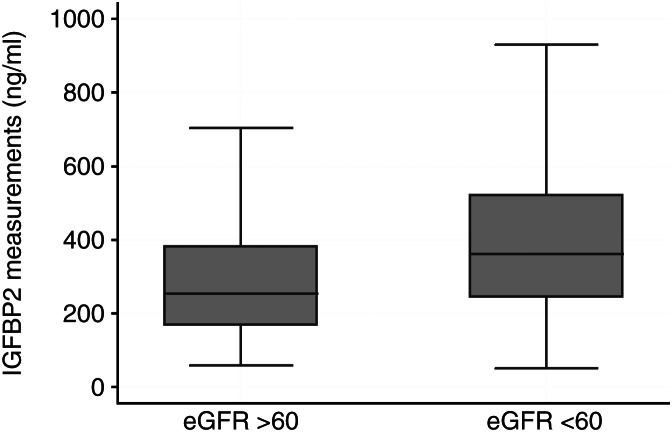
Box-plot representing plasma IGFBP2 concentration (ng/ml) in the study population classified according to baseline estimated glomerular filtration rate as above or below 60 ml/min per 1.73 m^2^. Horizontal line in the centre represents median IGFBP2 in each group, and box represents the interquartile ranges. Ends of whiskers indicate 5th and 95th percentiles. Outlying values not represented.

Concentrations of IGFBP1 (eGFR >60 ml/min per 1.73 m^2^, mean 2.6 ng/ml (95% CI 2.5–2.7); eGFR <60 ml/min per 1.73 m^2^, 2.9 (95% CI 2.7–3.1); *P*=0.0009 for log-IGFBP1) and IGFBP2 (eGFR >60 ml/min per 1.73 m^2^, mean 5.5 ng/ml (95% CI 5.4–5.6); eGFR <60 ml/min per 1.73 m^2^, 5.8 (95% CI 5.7–5.9); *P*<0.0001 for log-IGFBP2) were higher in the low eGFR group (<60 ml/min per 1.73 m^2^) as compared with the high eGFR group. There was no significant difference in IGF1, IGF2 or IGFBP3 between the two groups. The box and whisker plots in Fig. 1 illustrate median IGFBP2 levels in the low and high eGFR groups.

Three patients developed end-stage renal disease (eGFR <15 ml/min per 1.73 m^2^) during the study period. Two of these patients received haemodialysis, whereas one was treated conservatively. Of the 436 individuals (94%), 409 were alive at study completion in 2009. Of the 27 deceased subjects, 23 belonged to the low eGFR group at baseline.

### IGF proteins and baseline GFR

Each of the IGF proteins was studied individually as a predictor of baseline eGFR, with the study model adjusted for age, gender, diabetes duration, baseline measurements of systolic blood pressure, HbA1c and BMI, and the use of ACE inhibitors or angiotensin receptor blockers at study commencement as covariates.

High IGFBP2 concentration was associated with a low baseline eGFR in the analysis (*β*=−0.02 (95% CI −0.03 to −0.01), *P*<0.001). High IGFBP1 was also associated with low baseline GFR (*β*=−0.12 (95% CI −0.19 to −0.05), *P*=0.001). IGF1 (*P*=0.26), IGF2 (*P*=0.94) and IGFBP3 (*P*=0.29) were not associated with baseline eGFR. When all the five measured IGF proteins were taken together in the previous linear regression model, associations of eGFR with IGFBP1 (*β*=−0.09 (95% CI −0.17 to −0.02), *P*=0.008) and IGFBP2 (*β*=−0.02 (95% CI −0.03 to −0.01), *P*<0.001) were sustained, and high IGFBP3 was also associated with low eGFR (*β*=−2.4 (95% CI −4.2 to −0.53), *P*=0.012).

### IGF proteins and longitudinal renal function trends

Associations of IGF proteins with longitudinal trends in eGFR were analysed in mixed-effects regression models adjusted for age, gender, diabetes duration at study commencement, measurements of IGF1, IGF2, IGFBP1, IGFBP2 and IGFBP3, baseline concentrations of BMI, HbA1c and systolic blood pressure, and the use of ACE inhibitors or angiotensin receptor blockers during the study period.

**Figure 2:**
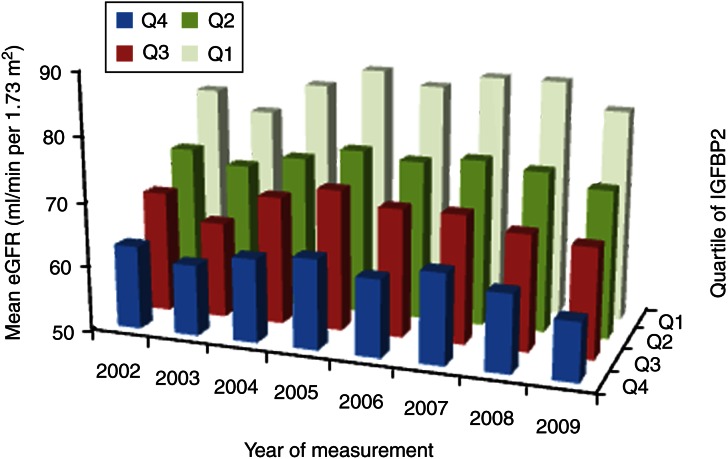
Graph representing yearly mean estimated glomerular filtration rates (eGFR) for the years 2002–2009 according to quartiles of baseline IGFBP2.

High IGFBP2 at baseline was associated with a decreased eGFR over an 8-year period from 2002 to 2009 (*β*=−0.02 (95% CI −0.03 to −0.01), *P*<0.001) in the above study model. Figure 2 illustrates mean eGFR values for each of the years from 2002 to 2009 according to quartiles of baseline IGFBP2. IGF1 (*P*=0.71), IGF2 (*P*=0.69), IGFBP1 (*P*=0.18) and IGFBP3 (*P*=0.17) were not associated with longitudinal eGFR trends in this study model.

## Conclusion

This study reports associations of high baseline IGFBP2 with adverse forward trends in renal function in type 2 diabetes. We found elevated IGFBP2 concentration to be associated with a longitudinal decline in GFR and an increase in proteinuria in Caucasians with type 2 diabetes.

IGFBP2 is the second most abundant circulating IGFBP (14), and has a greater affinity for IGF2 over IGF1 (15). Like other IGFBPs, it exerts biological effects that are both dependent on and independent of IGF1 and IGF2 (16, 17, 18). IGFBP2 concentrations correlate with insulin sensitivity, and low levels of this protein are a marker for the metabolic syndrome (19, 20, 21).

In the kidney, IGFBP2 expression has been demonstrated in the renal glomerulus, thin limbs of the loop of Henle and medullary collecting ducts, and may play an important role in renal podocyte development and maturation (3, 4, 22, 23, 24). IGFBP2 is preferentially expressed in the glomerulus, similar to the other IGFBPs: IGFBP1, IGFBP4 and IGFBP5. A study on STZ-induced diabetes rats indicated that renal mesangial concentrations of IGFBP2 and IGF2 are significantly increased early in the course of diabetes, along with local concentrations of IGF1 and IGFBP1 (25). However, other studies in STZ-diabetes rats have not demonstrated increased renal IGFBP2 mRNA levels in this model of diabetic nephropathy (9, 26, 27).

Previous studies have reported increased IGFBP2 concentrations in chronic renal failure (28). Poor glycaemic control in type 1 diabetes was associated with increased IGFBP2 proteolysis in albuminuric subjects (29). Associations of IGFBP2 with longitudinal trends in renal function have not been previously reported.

IGF2 is a major regulator of embryonic growth and development. While its postnatal roles have not been adequately clarified, it circulates at concentrations higher than that of IGF1.

IGF2 mRNA is widely expressed in renal tissue. Transgenic mice overexpressing *IGF2* demonstrate increased renal size (30). A study in STZ-induced diabetes rats reported an increase in serum and kidney IGF2 concentrations after the onset of diabetes, but without an increase in renal IGF2 mRNA expression (31). This would suggest that the increased renal IGF2 concentration was derived from the systemic circulation rather than produced within the kidney itself.

In a study that compared individuals with non-diabetic chronic kidney disease subjects with healthy controls, Frystyk *et al*. (32) reported significant increases in total serum IGF2 as well as IGFBP1 and IGFBP2 in those with nephropathy.

Glomerular hypertrophy is observed quite early in the pathophysiology of diabetic nephropathy. It is possible that an increase in IGFBP2 concentrations occurs in type 2 diabetes followed by an increase in local IGF1 and IGF2 concentrations in the renal glomerulus, very similar to the hypothesis postulated for IGF1 and IGFBP1 interaction in nephropathy. Given the affinity of IGFBP2 for IGF2, it may facilitate renal IGF1 and IGF2 accumulation. Longitudinal measurements of renal and systemic concentrations of IGF2 and IGFBP2 in diabetes and diabetic nephropathy will be needed to clarify which of these potential mechanisms results in the independent relation between higher circulating IGFBP2 and more adverse renal outcome over time.

Our study had some limitations. Being an observational ‘real-world’ study with clinical and biochemical tests instigated by the patients' usual healthcare providers, it was inevitable that investigations had not been performed on every patient annually. As albumin–creatinine ratios had not been measured in a number of patients (especially in the early years of the study), we have not reported associations of the IGF proteins with baseline and longitudinal trends in proteinuria. Some of the blood samples were collected from non-fasting subjects, which may have partly affected the results of analyses involving IGFBP1 (though IGFBP2 concentration is not affected by <72 h of fasting, and other IGF protein concentrations are also unaffected). Ideally we would have liked to measure IGF proteins at another time point to assess the change in IGF protein concentration in relation to changes in eGFR. While our study was aimed at a population with type 2 diabetes, it would be interesting to study whether the associations of IGFBP2 with eGFR are also true of a non-diabetic study population.

In summary, we report that IGFBP2 is a marker for deterioration in renal function in Caucasians with type 2 diabetes. The cellular activities of IGFBP2 in the kidney may have significant implications in diabetic nephropathy and research is required to elucidate whether there is a causative relationship between circulating IGFBP2 and progression of renal disease.

## Author contribution statement

R P Narayanan was involved in the design of the study, data collection and interpretation, and writing of the manuscript. B Fu assisted with the statistical analysis. A White provided antibodies for the IGF2 assay and reviewed the manuscript. K W Siddals, R L Oliver and J E Hudson performed the IGFBP1, IGFBP3, IGF1 and IGF2 assays. A Payton and W E R Ollier reviewed the manuscript. S G Anderson contributed to discussions. A H Heald was involved in the study design and reviewing the manuscript. J M Gibson was involved in study design and is the overall guarantor of the study.

## Figures and Tables

**Table 1:** Cardiovascular risk factor and medication profiles of study population at baseline and in 2009. Values expressed as arithmetic mean (s.d.).

	**Measurements in 2002**	**Measurements in 2009**
HbA1c (%)	8.0 (1.6)	11.8 (13.2)
HbA1c (mmol/mol)	64	99
eGFR (ml/min per 1.73 m^2^)	72 (18)	70 (27)
BMI (kg/m^2^)	31.8 (7.6)	31.0 (6.9)
BMI >30 kg/m^2^ (%)	55%	49.2%
Systolic BP (mmHg)	138 (18)	134 (20)
Diastolic BP (mmHg)	75 (11)	71 (10)
Total cholesterol (mmol/l)	4.6 (0.9)	3.9 (0.9)
HDL cholesterol (mmol/l)	1.2 (0.3)	1.3 (0.4)
Percentage of subjects on ACE inhibitors or ARBs	43.5 (in 2002)	67.8 (2002–2009)
Percentage of subjects on anti-hypertensive medication	54 (in 2002)	79.6 (2002–2009)
Percentage of subjects on metformin	58.5 (in 2002)	80.3 (2002–2009)
Percentage of subjects on sulphonylureas	36 (in 2002)	61 (2002–2009)
Percentage of subjects on insulin	29.5 (in 2002)	43.3 (2002–2009)
Percentage of subjects on statins	59 (in 2002)	85.5 (2002–2009)

eGFR, estimated glomerular filtration rate by the modification of diet in renal disease equation; BMI, body mass index; BP, blood pressure; HDL, high-density lipoprotein; ACE, angiotensin-converting enzyme; ARB, angiotensin-2 receptor blocker.

**Table 2:** Baseline characteristics of the study population grouped by glomerular filtration rate (GFR) above or below 60 ml/min per 1.73 m^2^ at study commencement. Clinical and biochemical characteristics of the study population at study commencement divided according to baseline estimated GFR as above or below 60 ml/min per 1.73 m^2^. Clinical variables described as arithmetic mean (s.d.) and IGF proteins expressed as median (interquartile range). Also described are *P* values on independent sample *t*-test analysis of the two groups. IGF1, IGF2, IGFBP1, IGFBP2 and IGFBP3 log-transformed for *t*-test analyses.

	**eGFR above 60 ml/min per 1.73 m^2^**	**eGFR below 60 ml/min per 1.73 m^2^**	***P* value**
Age	61.1 (10.5)	67.7 (9.3)	<0.0001
HbA1c (%)	8.2 (1.6)	7.7 (1.7)	0.018
HbA1c (mmol/mol)	66	61	
Body mass index (kg/m^2^)	32.0 (8.2)	31.0 (4.2)	0.28
Systolic BP (mmHg)	137 (17)	141 (20)	0.08
Diastolic BP (mmHg)	76 (10)	73 (11)	0.017
Total cholesterol (mmol/l)	4.6 (0.9)	4.5 (0.9)	0.83
HDL cholesterol (mmol/l)	1.2 (0.3)	1.2 (0.3)	0.73
IGF1 (ng/ml)	125 (64)	135 (87)	0.08
IGF2 (ng/ml)	584 (266)	617 (238)	0.22
IGFBP 1 (ng/ml)	11 (13)	15 (24)	0.0009
IGFBP 2 (ng/ml)	254 (214)	362 (276)	<0.0001
IGFBP 3 (mg/l)	4.3 (1)	4.3 (1.9)	0.34

BP, blood pressure; HDL, high-density lipoprotein; IGF, insulin-like growth factor; IGFBP, IGF-binding protein.
