# IGFBP2 is a biomarker for predicting longitudinal deterioration in renal function in type 2 diabetes

**DOI:** 10.1530/EC-12-0053e

**Published:** 2013-02-25

**Authors:** Ram P Narayanan, Bo Fu, Adrian H Heald, Kirk W Siddals, Robert L Oliver, Julie E Hudson, Antony Payton, Simon G Anderson, Anne White, William E R Ollier, J Martin Gibson

**Affiliations:** 1 Vascular Research GroupThe University of ManchesterManchester, M13 9PTUK; 2 School of Community Based Medicine, The University of ManchesterManchester, M13 9PTUK; 3 Centre for Integrated Genomic Medical Research, The University of ManchesterManchester, M13 9PTUK; 4 Cardiovascular Research GroupThe University of ManchesterManchester, M13 9PTUK; 5 Endocrinology and Diabetes, Faculty of Medical, Human and Life SciencesThe University of ManchesterManchester, M13 9PTUK; 6 Salford R&D, Salford Royal Hospital NHS Foundation TrustSalford, M6 8HDUK; 7 Department of Endocrinology and DiabetesSalford Royal Hospital NHS Foundation TrustSalford, M6 8HDUK

The author and journal apologise for an error in the above paper, which appeared in volume 1 part 2, pages 95–102. The error relates to some of the values in Table 1. The author's dataset for HbA1c in 2009 were in different units to data from 2002, which made the calculation of mean HbAb1c incorrect. The mean HbA1c (%) in 2009 should read 7.6 (1.5) and the mean HbA1c (mmol/mol) should read 60.

## Figures and Tables

**Table 1:** Cardiovascular risk factor and medication profiles of study population at baseline and in 2009. Values expressed as arithmetic mean (s.d.).

	**Measurements in 2002**	**Measurements in 2009**
HbA1c (%)	8.0 (1.6)	7.6 (1.5)
HbA1c (mmol/mol)	64	60
eGFR (ml/min per 1.73 m^2^)	72 (18)	70 (27)
BMI (kg/m^2^)	31.8 (7.6)	31.0 (6.9)
BMI >30 kg/m^2^ (%)	55	49.2
Systolic BP (mmHg)	138 (18)	134 (20)
Diastolic BP (mmHg)	75 (11)	71 (10)
Total cholesterol (mmol/l)	4.6 (0.9)	3.9 (0.9)
HDL cholesterol (mmol/l)	1.2 (0.3)	1.3 (0.4)
Percentage of subjects on ACE inhibitors or ARBs	43.5% (in 2002)	67.8% (2002–2009)
Percentage of subjects on anti-hypertensive medication	54% (in 2002)	79.6% (2002–2009)
Percentage of subjects on metformin	58.5% (in 2002)	80.3% (2002–2009)
Percentage of subjects on sulphonylureas	36% (in 2002)	61% (2002–2009)
Percentage of subjects on insulin	29.5% (in 2002)	43.3% (2002–2009)
Percentage of subjects on statins	59% (in 2002)	85.5% (2002–2009)

eGFR, estimated glomerular filtration rate by the modification of diet in renal disease equation; BMI, body mass index; BP, blood pressure; HDL, high-density lipoprotein; ACE, angiotensin-converting enzyme; ARB, angiotensin-2 receptor blocker.

